# Temporal Imaging of Live Cells by High-Speed Confocal Raman Microscopy

**DOI:** 10.3390/ma14133732

**Published:** 2021-07-03

**Authors:** Jeon Woong Kang, Freddy T. Nguyen, Niyom Lue

**Affiliations:** 1 Laser Biomedical Research Center, G. R. Harrison Spectroscopy Laboratory, Massachusetts Institute of Technology, Cambridge, MA 02139, USA; freddytn@mit.edu (F.T.N.); niyom.lue@ll.mit.edu (N.L.); 2 MIT Lincoln Laboratory, Lexington, MA 02421, USA

**Keywords:** Raman spectroscopy, Raman microscopy, cell analysis

## Abstract

Label-free live cell imaging was performed using a custom-built high-speed confocal Raman microscopy system. For various cell types, cell-intrinsic Raman bands were monitored. The high-resolution temporal Raman images clearly delineated the intracellular distribution of biologically important molecules such as protein, lipid, and DNA. Furthermore, optical phase delay measured using quantitative phase microscopy shows similarity with the image reconstructed from the protein Raman peak. This reported work demonstrates that Raman imaging is a powerful label-free technique for studying various biomedical problems in vitro with minimal sample preparation and external perturbation to the cellular system.

## 1. Introduction

Since it was first observed in 1928 [1], Raman spectroscopy has been broadly used as an analytical technique in various fields. Inelastically scattered Raman photons from the sample includes the “finger print” information about the sample. By analyzing Raman photons, chemical composition of the sample can be obtained qualitatively and quantitatively. Confocal microscopy has been utilized to obtain 3-D structural information from cells [2]. The confocal pinhole only accepts the light from the focus resulting in high axial resolution. Confocal microscopy was implemented with reflectance, fluorescence, or even with inelastically scattered photons including Raman [3] or Brillouin [4]. Combining confocal microscopy and Raman spectroscopy provides the three-dimensional chemical mapping of biological samples with high spatial resolution.

Puppels et al. first used confocal Raman microscopy for cell study in 1990 [3]. Although it showed promising results, confocal Raman microscopy has not been widely used for biomedical research, compared to the fluorescence microscopy, due to the low Raman conversion efficiency. For example, bright field imaging and quantitative phase microscopy (QPM) are the fastest, as the imaging speed is same as the frame rate of the camera. With advanced camera technology, more than 1000 fps (frames per second) is not uncommon. High-speed confocal fluorescence microscopy imaging speed is about 10 frames per second. High-speed confocal Raman microscopy takes about one minute per frame. In order to increase the imaging speed of Raman microscopy, line-scanning and multifocal Raman systems were developed [5,6]. Rather than mapping the whole cell, which can be highly time-consuming, researchers first identified morphologically relevant features of a cell under the bright-field imaging and subsequently measured the Raman spectra from the selected regions of interest [7]. Raman basis spectra were collected from this process and successfully utilized to develop clinical Raman devices and diagnosis software [8]. This two-step approach was very successful to balance between the high-resolution and high-speed data acquisitions.

Due to the advances of optics and electronic components such as spectrograph and detector, Raman mapping of cells became possible [9,10]. However, in the past, Raman imaging was not rapid enough to be considered as a practical tool for studying live cell dynamics. Tracking intracellular chemical composition during fast dynamics was challenging given these restraints. To address this limited speed issue, we have developed a custom-designed multi-modal microscopy system [11]. Instead of commonly used two-dimensional XY motorized stages, dual axis galvanometer mirrors perform fast scanning over the cells. On top of high-speed Raman imaging, this system also includes quantitative phase microscopy (QPM) to measure the two-dimensional optical phase delay of the sample. This multi-modal microscopy system has been applied for the malaria diagnosis by distinguishing hemoglobin and hemozoin distribution in the infected red blood cells [11]. This system has also been used to track the uptake dynamics of single walled carbon nanotubes to living macrophages with one frame per minute [12].

Here, we report the custom-built high-speed near-infrared (NIR) confocal Raman microscope system and demonstrate Raman imaging capabilities with various cell types. We present a convincing proof that the phase delay from QPM cellular image is mostly related to protein contents. Three types of live cells were studied to show the universal applicability of the system. Live HeLa cells (cervical cancer cells) were imaged to demonstrate the ability to acquire Raman hyperspectral images. Live HT-29 cells (colon cancer cells) were used to show the comparison between Raman and QPM images. Live RKO cells (colon cancer cells) were used to demonstrate the temporal imaging and tracking of different Raman bands with a four-minute temporal resolution. In the present study, Raman spectroscopy is shown as a viable option for real-time in vitro cellular imaging with high spatial and temporal resolution.

## 2. Materials and Methods

### 2.1. Setup

**Figure 1:**
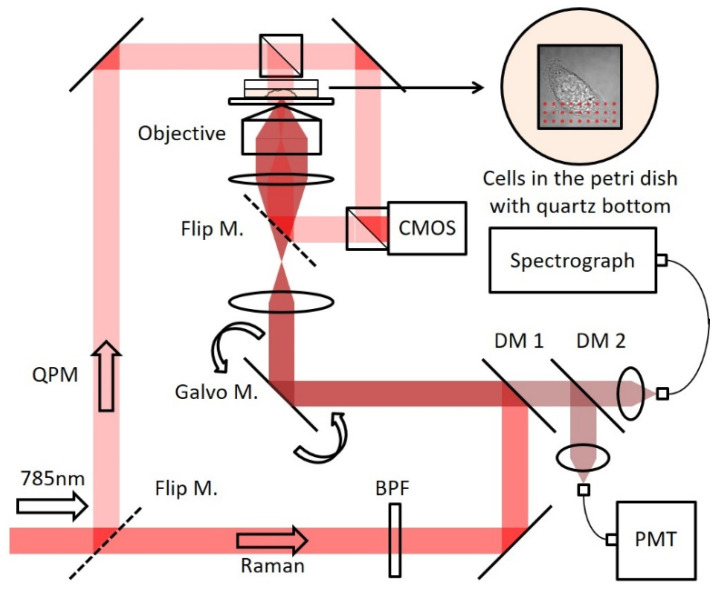
Schematic diagram of confocal Raman and quantitative phase microscopy system.

A Ti: Sapphire laser (3900S, Spectra-Physics, Milpitas, CA, USA) with 785 nm output wavelength was used as an excitation source of the custom-built NIR confocal Raman microscopy system (Figure 1) [11]. The collimated beam from the laser was cleaned by a laser line filter (BPF, LL01-785-12.5, Semrock, Rochester, NY, USA) and directed to the dual axis galvano mirrors. Laser beam was scanned in two dimensions by the galvano mirrors (CT-6210, Cambridge Technology, Bedford, USA). A high numerical aperture (NA 1.2) water immersion objective lens (Olympus UPLSAPO60XWIR 60X/1.20, Tokyo, Japan) focuses the laser light onto the cells and collects the scattered light from the cells, too. A piezoelectric actuator combined with a differential micrometer (DRV517, Thorlabs, Newton, MA, USA) controls the fine and coarse movement of sample focusing. A bright field imaging (75X magnification) of the cells was captured by inserting a flip mirror after the tube lens so that the sample plane can be imaged onto a CMOS camera (BCN-B050-U, Mightex, Toronto, ON, Canada). Raman photons generated from the cells pass through two dichroic mirrors (DM1: Semrock LPD01-785RU-25, Rochester, USA, DM2: Semrock LPD01-785RU-25 × 36 × 1.1, Rochester, USA). A step-index multi-mode fiber (Thorlabs M14L01, Newton, MA, USA) collects the Raman photons at the confocal pinhole location. Depending on the sample type, sampling volume can be increased or decreased by changing the collection fiber. The collected signal was delivered to the imaging spectrograph (Holospec f/1.8i, Kaiser Optical Systems, Ann Arbor, USA) and detected by a thermoelectric-cooled, back-illuminated, and deep depleted CCD (PIXIS: 100BR_eXcelon, Princeton Instruments, Trenton, USA). A single-mode fiber (Thorlabs P1-780A-FC-1, Newton, MA, USA) collects the reflected Rayleigh photons from the second dichroic mirror (DM2) and delivers them to a photomultiplier tube (PMT, H9656-20, Hamamatsu, Hamamatsu City, Japan) and a PMT controller (C7169, Hamamatsu, Hamamatsu City, Japan). This confocal reflectance setup provides co-registered images, which guide the optimal field of view for the Raman imaging. LabView 8.6 and a DAQ board (PCI-6251) from National Instruments (Austin, USA) control the experimental setup. Furthermore, Hilbert phase microscopy [13] was selected as a QPM modality. Based on Mach-Zehnder interferometry, the incident laser beam was divided into two and merged in front of the CMOS camera. From the intensity changes of the 1001 cm^−^^1^ peak during the axial scanning of the polystyrene bead, the axial resolution of the current configuration (50 µm core size collection fiber) was estimated to be 2.2 µm [14].

### 2.2. Cell Preparation

HeLa, HT-29 and RKO cells were cultured in Dulbecco’s Modified Eagle’s Medium (DMEM) with 10% fetal bovine serum (FBS) and 1% antibiotics. In order to maintain the viability of cells during the imaging, the selected cell was grown using custom-made petri dish with a quartz coverslip (043210-KJ, Alfa Aesar, Haverhill, MA, USA) at the bottom for at least 48 h before the imaging for good adherence of cells to the substrate. The quartz-bottom plates with the cells and culture media were imaged successfully without any damage to the cell over a period of 24 h.

### 2.3. Raman Mapping of Live Cells

60 mW of laser power was focused onto a micron size spot and delivered to the cells in fast raster scan. For the HeLa cells, 40 × 40 spectra were measured from 27 µm × 27 µm with a 1.0 s integration time. The total imaging time was about 27 min. For the HT-29 cells, 40 × 40 spectra were measured from 13 µm × 13 µm with a 0.5 s integration time. The total measurement time was approximately 14 min. For temporal imaging of the RKO cell, 40 × 40 spectra were measured from 36 µm × 36 µm at each time point with 0.15 s integration time. Ten Raman images were measured with a four minutes temporal resolution. Note that the integration time has been shortened with optimization and improvement of the system.

## 3. Results

One of the major advantages of this custom-built system is the flexibility of the system. The confocal sampling volume can be changed by changing the core size of the collection fiber. High-spatial resolution at the theoretical limit is not needed to characterize powder or liquid samples where integrated signal from larger volume is more critical. In that case, collecting as much Raman photons with a larger-core fiber is more relevant than collecting less signal while maintaining high resolution. When the sample has a fine structure, high-spatial resolution should be maintained. Cells fall into this category. Without this high spatial resolution, Raman imaging would not differentiate signals among different intracellular organelles. Although the focal plane is laterally positioned in the nucleus, the large focal volume integrates the Raman signal from the entire cell depth. Therefore, increasing the confocal sampling volume will decrease the Raman contrast. The axial resolution of the current system was configured to be 2.2 µm using a 50 µm core collection fiber.

**Figure 2:**
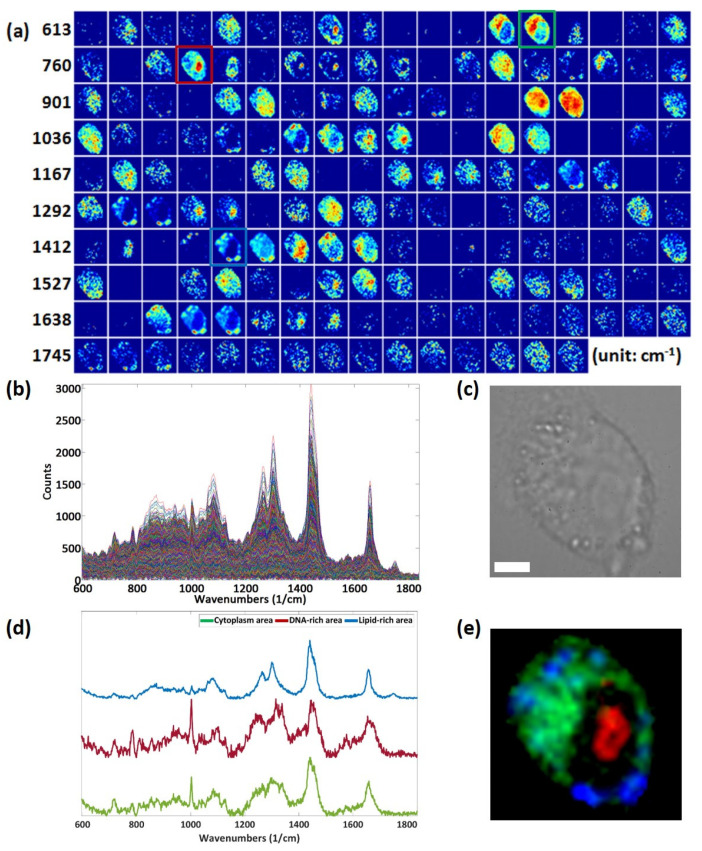
(**a**) Raman images of a live HeLa cell from various Raman bands between 613 cm^−1^ and 1825 cm^−1^. Different Raman bands show different distributions throughout the cell. (**b**) A total of 1600 overlaid Raman spectra (40 × 40 spectra) from a single cell. (**c**) Bright-field image of live HeLa cell used in the experiment. (Scale bar: 5 µm). (**d**) Representative spectra from cytoplasm, nucleolus, and lipid droplets. (**e**) Pseudo-color image reconstructed from 785 cm^−1^ (red), 720 cm^−1^ (green), and 1450 cm^−1^ (blue) Raman bands.

The live HeLa cell was imaged and analyzed using single peak analysis. Raman images were reconstructed from different Raman bands between 613 cm^−1^ and 1825 cm^−1^ (Figure 2a). Images reconstructed from single Raman bands with 15 cm^−1^ bandwidth reveal high-resolution images and biochemical maps. Specifically, the 720 cm^−1^, 785 cm^−1^, 1004 cm^−1^, and 1450 cm^−1^ peaks correlate to the cytoplasm, DNA, protein and lipid distributions, respectively [15]. This information is well correlated to the bright-field image in Figure 2c. Since the protein signal is uniformly spread over the entire cell volume, this signal was excluded and a pseudo-color image was generated from three other Raman bands corresponding to the cytoplasm, DNA, and lipid Raman signals. In Figure 2e, the red color represents 785 cm^−1^ (DNA) and is localized to the cell nucleus. Green and blue represents 720 cm^−1^ (cytoplasm) and 1450 cm^−1^ (lipids), respectively. Although Raman imaging cannot compete with the imaging speed and resolution of fluorescence imaging, Raman imaging can be performed without any staining, sample preparation, or external perturbation to the cell. In summary, Raman imaging and mapping can simultaneously track multiple chemical components of the live cell in vitro.

**Figure 3:**
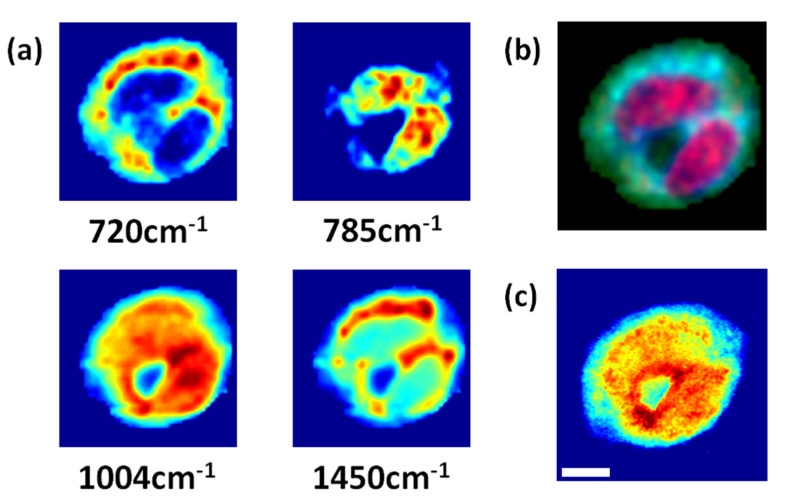
(**a**) Four Raman images reconstructed from live HT-29 cell (40 × 40 pixels). (**b**) Pseudo-color image reconstructed from 785 cm^−1^ (red), 720 cm^−1^ (green), and 1450 cm^−1^ (blue) Raman bands. (**c**) Quantitative phase microscopy image of the same cell. (Scale bar: 3 µm) Optical phase delay measured from QPM is similar to the reconstructed image from the 1004 cm^−1^ Raman peak.

Phase contrast microscopy is the most commonly used method to see detailed cellular morphology without staining the cell. Recently, quantitative phase microscopy (QPM) with a full field interferometric measurement, has become a popular tool to study cellular dynamics and morphology [16,17]. However, since optical phase delay is an indirect measurement of chemical information, the interpretation of QPM images is not necessarily trivial. Live HT-29 cells were used to see the most relevant chemical component for phase contrast imaging. For this study, four Raman images corresponding to the 720 cm^−1^, 785 cm^−1^, 1004 cm^−1^, and 1450 cm^−1^ peaks (Figure 3a) were compared to the QPM image (Figure 3c). Among the four images, the Raman image from the 1004 cm^−1^ peak correlates the closest with the QPM image. This result confirms recent works in QPM that the phase delay in cells mostly represents protein distribution [18]. Therefore, protein related cellular phenomena are a preferable target for QPM techniques.

**Figure 4:**
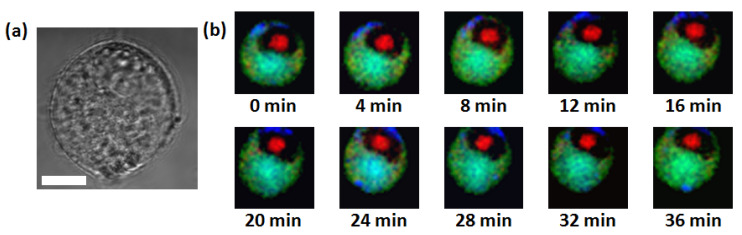
(**a**) Bright field image of live RKO cell used in the experiment. (Scale bar: 10 µm) (**b**) Temporal Raman images of live RKO cell (40 × 40 pixels) with four-minute temporal resolution. Red, green, and blue represent 785 cm^−1^, 720 cm^−1^, and 1450 cm^−1^ Raman bands, respectively.

Temporal imaging capability was demonstrated by measuring multiple Raman images from live RKO cells. Ten Raman images (40 × 40 pixels) were acquired with 0.15 s integration time. With no time delay between frames, 16,000 (40 × 40 × 10) Raman spectra were acquired over a 40 min time period. 720 cm^−1^, 785 cm^−1^, and 1450 cm^−1^ Raman bands were selected for constructing the temporal pseudo-color images (Figure 4b). Intracellular chemical changes such as DNA degradation, protein contents change, or lipid droplet formation can be monitored with a temporal resolution of four minutes.

## 4. Discussion

Here, we have demonstrated the temporal high-resolution Raman imaging of live cells. Intracellular distributions of biochemically relevant components, such as DNA, protein, and lipids, were successfully monitored with a temporal resolution of four minutes and an axial resolution of 2.2 µm. Through the design, assembly, and optimization of the confocal Raman system using the latest state-of-the-art components, Raman imaging of cells is possible and a practical method for studying dynamic cellular systems.

As reported, confocal Raman imaging can become a powerful method for monitoring the status of the cell and associated chemical changes in real-time. Since the cells do not need to be stained or altered for Raman imaging, live cell dynamics can be observed with minimal or no external perturbation. Many biological applications will benefit from these label-free quantitative chemical imaging capabilities. Few ongoing examples include studying the effects of cellular uptake of particles [12,19], effects of drugs/UV radiations [14,20], and cellular growth and development.

## Data Availability

The data presented in this study are available on request from the corresponding author.
